# Charity by Proxy or Semi-Charity and Its Disastrous Effect upon the Physician1Address by the retiring president at the annual meeting of the Buffalo Academy of Medicine, June 11, 1901.

**Published:** 1901-08

**Authors:** Marcell Hartwig

**Affiliations:** Buffalo, N. Y.


### Charity by Proxy or Semi-Charity and Its Disastrous Effect Upon the Physician.[1]

By MARCELL HARTWIG, M. D., Buffalo, N. Y.

REMOTA causa tollit effectus. If we desire to eradicate an evil we have to search for its foundation. The glamor about dispensary abuse will soon become reinforced with fog-horn rumbling. The time is drawing near when the medical profession will begin to realise how vitally it is being imposed upon by the hospital. The iniquity is growing daily. The physician is becoming the tool of the hospital instead of being its manager. If some people are desirous of aggrandisement and found a hospital, or if they are really charitable, they but need to wink, and like a mob of hungry wolves, a bunch of physicians are on hand and consider it an honor to be permitted to offer their services gratuitously, all in the more or less substantiated hope to obtain, indirectly, patients, fame and name. Few go for the scientific opportunities of the hospital. Very nice and laudable, how ready they offer their services to help the unfortunate fellow beings; very admirable how the founders give their means and energies for such honorable undertaking. The trumpets of the daily press sound their praises. But how does it look behind the curtain? The maintainance of the really poor is paid for by the city and county, while both lose the control.

The well-to-do receive special care and accommodation for ludicrously low pay. Any hotel would not maintain them for twice the price while they are in good health. Where does the charity thus go, *id est*, that part of the expense of the hospital which the generous givers, above and beyond the public assistance, furnish? To the pay patients. Who reaps the benefit? The attending physicians. The others remain in the cold. And doubly cold they have to suffer, inasmuch as at times they are forced to recommend to their patients for their best interests to seek the hospital, and here they lose their patients into the hands of others; at least very often.

Meanwhile, the attending physicians may displease the hospital managers. Bang! They are fired, perhaps after having done years of charitable work and just begun to reap the benefits of their assiduous labors. Are such conditions worthy of an

---

1. Address by the retiring president at the annual meeting of the Buffalo Academy of Medicine, June 11, 1901.

enlightened profession, which is certainly willing to do proportionately as much charity as the wealthy neighbors?

The development of medical science is making the hospital more and more a frequent requirement for the best management of patients, and thus grows the dissatisfaction of physicians, a regular circulus vitiosus.

But the patients suffer as well—not those in the hospital, but those outside of it. The loss of patients—transferred to the hospital and attended gratuitously—forced the physicians to demand bigger fees. The hospital physicians, doing a lot of work gratuitously, demand for their prestige still higher prices, and as every patient cannot go to a hospital, the middle classes with limited incomes are in a sore plight to find decent physicians for fair prices. Thus, even in this field, the trend of the times to develop two classes—millionaires and paupers—is fostered.

Another way the hospital aids this economic movement lies in the wholesale agreement, tacit or actual, between the hospital and the large employers. Where the employer is obliged to take care of his workman, accidentally hurt in employ, he finds it much cheaper to send him to the hospital than to have him attended to at home. And, where the employer is in the board of hospital governors, he has a good chance to find a free bed. At all events, he has partial charity extended to a man, where otherwise he would be obliged to pay in full. He does not pay the physician; the hospital no taxes. Where is the remedy? I see only one, and that is that the hospital must be owned by the physicians, themselves, in common. Every one must have the same privileges; every patient the choice of his doctor. Such ideal condition cannot be brought about except every physician would be part owner of the hospital, with equal voice to every other. But, how should such condition be brought about, as every physician is not equally rich, and the owner of a lot of shares would soon oust the others? I see only one way. A new form of share-companies would have to be legalised, where every owner of one share would have as much voice as one of a hundred shares, though profits, if any, would have to remain proportionate to shares. Such new share companies and their legal status should be discussed by the lawyers. For my part, I add that such a form of share company is, to my view, one of the few sound means to counteract the dangerous tendency of the times, to wipe out the middle classes and construct a plutocratic oligarchy. If a man's voice in the government of the state is as good as another's, why should such a thing be impossible in business.

We physicians are in more than one sense laboring men and with the enlarged field of vision at our command, with our intimate knowledge of human affairs among the lowly as amongst the affluent, with our proverbial warm feeling and mostly genuine sympathy with the afflicted, ought to march in the vanguard when the means are considered to do the greatest good to the greatest number; and as one of those means I propose, the introduction and labor for the construction of the above mentioned share-companies. By thus benefiting ourselves we may yet form the example for a new means of benefiting the masses. At all events, I thought of not being able to proffer a more worthy object for an animated discussion to-night.

*Salus publica suprema lex esto.*

884 MAIN STREET.

